# Proton Magnetic Resonance Spectroscopy of the motor cortex reveals long term GABA change following anodal Transcranial Direct Current Stimulation

**DOI:** 10.1038/s41598-019-39262-7

**Published:** 2019-02-26

**Authors:** Harshal Jayeshkumar Patel, Sandro Romanzetti, Antonello Pellicano, Michael A. Nitsche, Kathrin Reetz, Ferdinand Binkofski

**Affiliations:** 1 Division of Clinical Cognitive Sciences, Department of Neurology, RWTH Aachen University, 52074 Aachen, Germany; 2 Department of Neurology, RWTH Aachen University, 52074 Aachen, Germany; 3 Institute of Neuroscience and Medicine (INM-4), Research Center Jülich GmbH, 52425 Jülich, Germany; 4 Leibniz Research Centre for Working Environment and Human Factors, Department of Psychology and Neurosciences, 44139 Dortmund, Germany; 5 Department of Neurology, University Medical Hospital Bergmannsheil, Bochum, Germany

## Abstract

Anodal transcranial direct current stimulation (tDCS) over the primary motor cortex (M1) has been reported to increase the firing rates of neurons and to modulate the gamma-aminobutyric acid (GABA) concentration. To date, knowledge about the nature and duration of these tDCS induced effects is incomplete. We aimed to investigate long-term effects of anodal tDCS over M1 on GABA dynamics in humans. Repeated magnetic resonance spectroscopy (MRS) was employed to measure relative GABA concentration in M1 for approximately 64 minutes after stimulation. The study was performed on 32 healthy subjects. Either anodal or sham tDCS were applied for 10 minutes with the active electrode over the left M1 and the reference electrode over the right supra-orbital region. Pre and post-tDCS MRS scans were performed to acquire GABA-edited spectra using 3 T Prisma Siemens scanner. GABA signals showed no change over time in the sham tDCS group, whereas anodal tDCS resulted in a significant early decrease within 25 minutes after tDCS and then significant late decrease after 66 minutes which continued until the last test measurements. The late changes in GABA concentration might be related to long-term plasticity mechanism. These results contribute to a better understanding of the neurochemical mechanism underlying long-term cortical plasticity following anodal tDCS.

## Introduction

Transcranial direct current stimulation (tDCS) is a non-invasive stimulation technique that allows for the modulation of cortical excitability in humans. Animal studies^1^, as well as studies on humans^2,3^ have shown that anodal tDCS leads to cortical facilitation, that is increased overall excitability, and increases spontaneous firing rates of cortical neurons. The facilitation effect of anodal tDCS has also been associated with improved cognitive and behavioral skills as reported in healthy participants and patients^4–6^.

So far, short- and long-term sustainability (i.e., after-effects) of increased excitability have been observed to depend on the duration of tDCS^2^. Previous neurophysiological studies have shown that tDCS application between 5 to 7 minutes over M1 led to short-term effects (increase of transcranial magnetic stimulation (TMS) elicited Motor Evoked Potential (MEP) amplitudes) for no longer than 5 minutes, whereas 9 to 13 minutes of stimulation have been found to induce a long lasting increase of excitability for up to 90 min^2,7^. Moreover, the increased duration of cortical excitability has been further demonstrated by applying two consecutive tDCS sessions on the M1 of healthy humans^8^. The second stimulation was applied without an interval, during the after-effects of the first stimulation, or after the after-effects of the first stimulation had vanished. The during after-effects condition resulted in an initially reduced, but then relevantly induced l-LTP like plasticity through prolonged excitability enhancement in M1. This increase of excitability is supposed to be associated in humans to the reduction of gamma-aminobutyric acid (GABA)-driven inhibition, as displayed in motor cortex studies^9,10^. GABA is an inhibitory neurotransmitter and has a prominent role in human motor learning^11,12^. However, knowledge about the long term effects of tDCS on GABA neurotransmission is incomplete. Until now, only few studies have investigated the role of GABA in cortical plasticity following anodal tDCS. Effects on cortical plasticity as a function of GABA modulation have been observed up to 20 minutes in healthy participants^13^ and up to 25 minutes in chronic stroke recovery patients^14–16^. More recently, a study using a similar stimulation protocol displayed a depletion of ATP and Phosphocreatine over M1 for a longer time, that is approximately 90 minutes^17^. As ATP and Phosphocreatine are indexes of energy consumption in brain tissues, this localized decrease of their concentration has been associated to a long-term effect of increased energy consumption after anodal tDCS.

However, whether anodal tDCS can also produce long-term plasticity effects associated to reduced GABA concentration remains untested. In the present study we aimed at better understanding the mechanism of neural plasticity. Hence, we measure the effects of anodal tDCS on GABA concentration for one hour and more.

## Results

**Figure 1:**
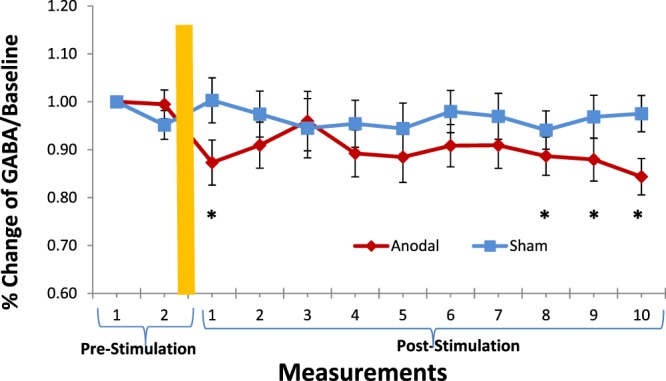
Mean GABA percentages of concentration for anodal and sham stimulation groups with 16 subjects per group. The graph shows a significant decrease of GABA concentration in the anodal stimulation group at first, eight, ninth and tenth post-stimulation measurements with respect to the first pre-stimulation measurement condition (*p < 0.05). Error bars reflect standard error of the mean.

Statistical analysis was conducted with SPSS (version 23.0 Armonk, NY, USA) with a *p* = 0.05 set as the threshold for significance. A mixed-design Analysis of Variance (ANOVA) was performed on GABA concentration with *Stimulation* (sham vs. anodal) as the between-participants factor and *Measurement* (12 measurements: 2 pre-stimulation and 10 post-stimulation measurements) as the within-participants factor, respectively. Paired-sample *t*-tests (confidence interval 95%) were conducted as post-hoc analyses of factors interaction. Partial eta-squared (η^2^_p_) was calculated within the ANOVA as measure of effect size. An open-source tool was used to compute Cohen’s *d*_*z*_ effect size for the *t*-tests (https://webpower.psychstat.org/models/means01/effectsize.php). Figure 1 shows the time evolution of the normalized GABA concentration level induced by tDCS at different measurement times. The main effect of Stimulation was not significant [*F*_(1, 30)_ = 1.157, *p* = 0.291 *p*_rep_ = 0.644 η^2^_p_ = 0.037]. The main effect of Measurement was significant [*F*_(11, 330)_ = 2.378, *p* = 0.008, *p*_rep_ = 0.961 η^2^_p_ = 0.073]. Remarkably, the interaction Stimulation x Measurement was significant [*F*_(11, 330)_ = 2.249, *p* = 0.012 *p*_rep_ = 0.950 η^2^_p_ = 0.070]. For the sham and anodal group, paired-sample *t*-test comparisons were performed between the first pre-stimulation measurement condition and each of the subsequent measurements (i.e., second pre-stimulation and first to tenth post-stimulation conditions). For the sham group, GABA concentration did not change significantly from the first pre-stimulation measurement to all the following measurements [*ts*_(15)_ ≤ 1.715 *p*s > 0.05]. For the anodal tDCS group, relative to the first pre-stimulation measurement, GABA concentration did not change significantly at the second pre-stimulation [*t*_(15)_ = 0.347 *p* = 0.733 *d*_*z*_ = 0.087] but decreased significantly at the first post-stimulation measurement [*t*_(15)_ = 2.226 *p* = 0.042 *p*_rep_ = 0.889 *d*_*z*_ = 0.556]. Thereafter, GABA concentration returned close to baseline level from the second until the seventh post-stimulation measurements [*t*s_(15)_ ≤ 1.776 *p*s > 0.05 *d*_*z*_s ≤ 0.444], and then further decreased significantly at the eighth to tenth post-stimulation measurements [*t*(15) = 2.514 *p* = 0.024 *p*_rep_ = 0.922 *d*_*z*_ = 0.629; *t*_(15)_ = 2.410 *p* = 0.029 *p*_rep_ = 0.912 *d*_*z*_ = 0.602; and *t*_(15)_ = 3.483 *p* = 0.003 *p*_rep_ = 0.980 *d*_*z*_ = 0.871] (Fig. 1). Thus, while in the sham group GABA concentration stayed at the pre-stimulation baseline level across all post-stimulation measurements, data from the anodal group suggested a decreasing pattern of GABA concentrations across different time points. GABA concentration first showed a decrease at the early first post-stimulation measurement, and then a more consistent decrease (approximately for 34 minutes) at the later eighth to the tenth post-stimulation measurements. To rule out the possibility that the observed GABA decrease depended on longitudinal changes in the fit of the MRS data specific to the stimulation group, we performed a mixed ANOVA on Cramér-Rao-Lower-Bounds (CRLB) values. This analysis revealed no significant effect of Stimulation [*F*_(1, 30)_ = 0.387, *p* = 0.539, *p*_rep_ = 0.473 η^2^_p_ = 0.013], Measurement [*F*_(11, 330)_ = 1.581, *p* = 0.103, *p*_rep_ = 0.808 η^2^_p_ = 0.050], and Stimulation x Measurement interaction [*F*_(11, 330)_ = 1.566, *p* = 0.107, *p*_rep_ = 0.804 η^2^_p_ = 0.050], thus supported the hypothesis of decreased GABA concentrations produced by anodal stimulation.

## Discussion

The purpose of this study was to explore the neurochemical mechanism underlying after effects of anodal tDCS and their long-term duration by means of repetitive MRS measurements of GABA levels. Our results suggest a drop in GABA levels within 25 minutes following anodal stimulation (at first post-stimulation measurement) which is consistent with previous reports^13^. Furthermore, our results also suggest late after effects of anodal tDCS, which emerged about 66 minutes (i.e. at the eighth post-stimulation measurement) and remained stable until about 90 minutes (at the last post-stimulation measurement). Our finding that anodal, but not sham, tDCS delivered to the motor cortex leads to a significant decrease in MRS-GABA within the stimulated region, shows that the excitatory effects of anodal tDCS would be associated with a modulation of GABAergic interneurons^12,18,19^, which might be due to downregulation of the enzyme GAD-67 activity^20,21^. Excitatory effects of anodal tDCS seem to trigger compensatory changes in neuronal excitation. One example of such synaptic homeostasis is the down regulation of the enzyme GAD-67, which critically controls GABA synthesis while likely gating glutamatergic plasticity of the M1. Hence, enhanced glutamatergic activity following anodal tDCS might be one of the mechanism gated by GABA down regulation. The significant reduction of cortical GABA seems not to be involved only in early phase but also late phase effects after the end of tDCS over a period of approximately 90 minutes. It would occur (i) in the form of short-time plasticity after the end of tDCS within 25 minutes after the end of tDCS and (ii) a consolidated long term effect after 66 minutes following tDCS which continues until the end (90th minutes) of the last measurement. Noteworthy, the time-line of GABA depletion is similar to the reduction pattern of high-energy phosphates as demonstrated by phosphorus spectroscopy with a similar anodal tDCS protocol^17^. Such a similarity between the time course of reduced GABA concentration and high energy phosphates concentration suggests that anodal tDCS would be able to induce an active neural process, most probably gated by GABA-ergic plasticity as one source as seen in our results.

A study from dyke and colleagues^22^ reported that concentrations of GABA measured by MRS, and GABA-mediated physiological inhibition indexed by 3 ms SICI (short-interval intracortical inhibition) are unrelated; which replicated previous results for 3 ms SICI^23^ and 2.5 ms SICI^24^. The physiological mechanism underlying 2.5 ms and 3 ms involves post-synaptic inhibition mediated through GABA-A receptors^25^. Lack of correlation between GABA measured by MRS and GABA indexed by SICI suggests that one of the possible source of MRS-GABA modulation following anodal stimulation in our study might be due to extracellular GABA levels and may not be associated with synaptic transmission^26,27^. However, a study from Stagg and colleagues^24^ reported a significant correlation between MRS-GABA and 1 ms SICI effects, which may be related to refractory period of inter-neurons^28^ or synaptic processes^29^. However, the exact mechanism underlying correlation of MRS-GABA and 1 ms SICI remained unclear.

The current study suggests first evidence for a long term after effects on GABA modulation following anodal tDCS. Results deliver a first move towards the understanding of long term effects of anodal tDCS and raise the question on how long the GABA concentration takes to return to baseline values. Our data do not provide a clear explanation on the return of GABA to baseline following depletion. Longer duration investigations including several measurements (e.g across more than three hours) might shed some light on the return of GABA concentration at baseline levels following significant decrease after anodal tDCS. The GABA and glutamate are closely linked in the human brain and this relationship has been confirmed by MRS studies^13^. However, due to technical reasons the GABA edited MEGA PRESS sequence does not allow us to separate glutamate and glutamine and reliably quantify the composite measure of glutamate and glutamine (Glx). A study, for example at 7 T MRI scanner, directly investigating long term glutamate modulation has yet to be performed to explore more about long term plasticity-like mechanism.

Although the implementation of a cross-over design (i.e., one group having both anodal and sham stimulations) would have provided some increased statistical power, it could have generated cumulative excitability effects^30,31^ and would have exposed the participants to experience different skin reactions or sensations^32^ between the two sessions, thus introducing the risk of significant biases on the final data. For this reason, we chose a parallel design to avoid any carry over effect from active anodal to sham stimulations and vice versa.

Chew and colleagues^33^ investigated cortical excitability after M1 anodal tDCS (10 min duration, 16 cm^2^ target reference electrodes) and did not observe a main effect of intensity; however no sham condition was tested. Intra-individual reliability of 0.5 over 30 minutes following stimulation was reported (poor ICC (2,1) = −0.5), and participants responded strongly to 0.2 and 2 mA, only. Moreover, reliability of prefrontal tDCS-induced resting state function MRI modulation (20 min 35 cm^2^ target/reference 2 mA, bipolar stimulation) was investigated: low reliability was observed for active tDCS compared to baseline and sham groups^34^. A study investigating 1.0 mA anodal tDCS (13 min duration, 35 cm^2^ target/reference electrodes) reported good intra-individual reliability over the first 30 min (ICC(2,1) = 0.565), although measurements obtained during the 30 min afterwards showed poorer reliability (ICC(2,1) = − 0.028)^35^. One further study (15 min duration, 35 cm^2^/100 cm^2^ target/reference) investigated intra-individual reliability in 1.0 mA anodal tDCS and showed stronger reliability, both over early and late measurement periods (ICC(2,1) = 0.74 and 0.64, between 0–30 and 60–120 min, respectively)^36^. Methodological differences are present between these studies, such as sample sizes and stimulation parameters, which may promote to different findings. Indeed, it is known that MEP amplitude change is affected by the size of the electrode^37–39^, as well as by the duration of the tDCS^40,41^. Moreover, we have also investigated the fit quality of the MRS data specific to the stimulation group because the decrease in GABA levels following anodal stimulation might results from intra-individual changes in the fit quality of GABA levels. The statistical analysis, as mentioned in the result section, revealed neither significant main effects of Stimulation and Measurement, nor their significant interaction. Another possible reason for low reliability may be due to elevated drowsiness associated with participants during the MRS measurements which may affect cortical excitability and corresponding GABA modulation. In an effort to regulate these factors, all participants in the study were informed about the measurement duration before the start of the MRS measurement and their level of vigilance was monitored during the whole experiment. Furthermore, as also different time lags between sessions may affect vigilance and attention of the participants and bias the results, we kept the day time of measurements rather constant between sessions.

Regarding application-relevant aspects of the results of this study in stroke rehabilitation, it is of much interest to foster motor learning to aid the development and implementation of adjunctive therapies. Thus, reduction in GABA concentrations via tDCS might gate glutamatergic plasticity, which could be used to investigate motor learning effects. Our experimental approach might furthermore provide a methodological approach to non-invasively investigate if patients with neurodegenerative diseases express abnormal forms of long-lasting plasticity in M1.

It is also important to understand the interaction of tDCS and GABA with pharmacological treatment in clinical practice to a larger extent. Given that GABAergic cortical inhibitory interneurons play a role in the early stage of Alzheimer’s disease^42^, modulation of these GABA interneurons by tDCS could be a potential disease-modifying mechanism for restoring working memory and cognition. Furthermore, the transferability of results from motor cortex experiments to other critical targets, such as the dorsolateral prefrontal cortex, is unclear at present. Hence, further research is needed to determine if mechanisms found in studies investigating M1 are also relevant to other brain target regions. So far, GABA has not been widely explored with regard to tDCS induced plasticity, but it might be an important contributor to cognitive neuroscience research which deserves higher consideration.

We have demonstrated that anodal tDCS induces long-lasting effects of anodal tDCS on GABA concentrations, and to measure the corresponding early and delayed GABA changes at the molecular level for nearly one and half hour. Here we propose long term GABA reduction as one potential elements contributing to local cortical changes in M1 following anodal tDCS, which might gate glutamatergic plasticity.

## Materials and Methods

### Participants

Thirty-two healthy volunteers (mean age 26 ± 4, range 22–30 years) with no history of neurological or psychiatric diseases participated in this single blind randomized control study. Participants were assigned to two groups by counterbalancing the sex, and matching their age as much as possible between the groups. Sixteen participants were assigned to the *Anodal stimulation group* (8 females, mean age = 27.25, SD = 6.48; 8 males, mean age = 25.63, SD = 3.58), and sixteen to the *Sham stimulation group* (8 females, mean age = 26.75, SD = 3.92; 8 males, mean age = 25.25, SD = 3.28), that is, the control group for possible changes of GABA concentrations unrelated to the anodal stimulation. All participants were right handed as assessed by the Edinburgh Handedness Inventory (Oldfield, 1971). All assessments were conducted at the Division of Clinical Cognitive Sciences of the RWTH Aachen University, Germany, and after participants gave their written informed consent for participation in this study, which was approved by the ethics committee of the Medical Faculty-RWTH Aachen University. All methods were performed in accordance with the relevant guidelines and regulations of the institution.

### Transcranial direct current stimulation (tDCS) and sham stimulation

A DC-Stimulator (neuroConn GmbH, Ilmenau, Germany) delivered 1 mA of electric current to the brain *via* two rubber electrodes (5 × 7 cm) covered by saline-soaked (0.9% NaCl) sponges. One electrode was positioned 5 cm lateral from Cz and 2 cm anterior to the mid-pre-central position of the left hemisphere, to stimulate left primary motor cortex (M1), and the other over the contralateral supraorbital ridge using convention of EEG 10/20 system. To ensure accuracy, during the positioning of the electrodes, we adhered to EEG 10–20 system; a method that provides reproducible and consistent placement of electrodes for different head sizes^43,44^, and kept a distance of not less than 6 cm between the sponges. Indeed, tDCS with electrode covering area of 35 cm^2^ is not focal, and variation of displacement of the electrode by 1 cm should not have significant impact on current flow^45^.

For real (anodal) stimulation, the current was ramped-up for 10 s, kept constant at 1 mA for 10 min, and finally ramped down over a period of 10 s. For sham stimulation, the current was ramped up for 10 s and then immediately ramped-down and kept off for the next 10 minutes.

### MRS acquisition

**Figure 2:**
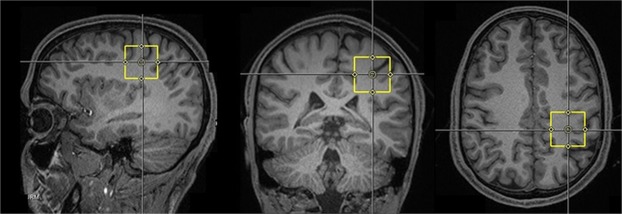
Representative sagittal, coronal and axial T1-weighted MRI brain images of a subject depicting the 3 × 3 × 3 cm voxel (in yellow) within the primary motor cortex.

All measurements were performed with a 3 T Siemens PRISMA MR scanner (Siemens, Erlangen, Germany). The system was equipped with a dockable patient bed and participants were asked to lay at rest on it throughout the experimental session. Each experimental session started with the acquisition of sagittal T1-weighted images, which were used to carefully place a 3 × 3 × 3 cm voxel of interest within the hand area of the left M1 as shown in Fig. 2. Voxel size of 3 × 3 × 3 cm is frequently used to acquire MRS data reliably and offer trade-off between localization and data quality^46^. Then, a careful shimming procedure was performed using a FASTEST map sequences. In order to achieve good field homogeneity, linewidths of water at full width at half maximum (11 ± 1 Hz in M1) was obtained by shimming maximum three times before first pre- and post-stimulation measurement. To assess the creatine and N-acetylaspartate acid (NAA) line widths, a standard PRESS sequence was used to acquire an unedited spectrum with 32 averages. A MEGA-PRESS editing sequence^47^ (with parameters repetition time = 2000 ms, echo time = 68 ms, averages = 96 and editing pulses centered at 1.9 and 7.5 ppm on each alternate scan) was used to acquire GABA spectra. The acquisition and analysis protocol used in this study followed recently published guidelines for GABA MRS at 3 T using MEGA-PRESS^46^. Each acquisition took approximately 6 minutes and 25 seconds.

It has been observed that within-participants stimulations over consecutive days can cause cumulative and larger excitability effects^30,31^. No standard guidelines have been established yet on the amount of time that should be left between tDCS sessions to ensure that any stimulatory effects have washed out^48^. For these reasons, stimulations were administered between participants with separate stimulation groups. Furthermore, a cross-over design can bring the participants to notice different skin reactions or sensations^32^ between the two sessions, thus potentially introducing significant biases on the final data.

**Figure 3:**
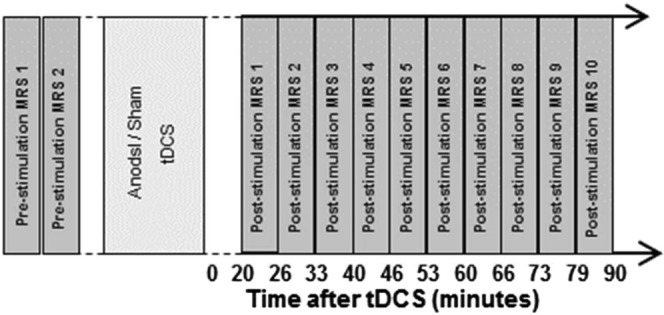
Layout of the experimental procedure depicting the GABA spectroscopy measurements performed before (pre-stimulation) and after (post-stimulation) the application of transcranial direct current stimulation.

Two baseline GABA measurements were performed before tDCS (*pre-stimulation* measurements). Successively, subjects were asked to hold their position, while the patient table was undocked from the scanner and moved outside of the magnet room. This allowed to minimize the movements of the participant’s head between the pre- and post-stimulation MRS measurements. The participants were asked not to move while the stimulation electrodes were placed on pre-marked areas of the head. The stimulation was then started, and after 10 minutes, electrodes were removed carefully from the head of the participants, and the table docked again to the MR scanner. Electrode removal from participant head, participant table docking in the scanner, running localizer, anatomical acquisition, voxel replacement and shimming were performed between the end of tDCS and the start of the first post-stimulation measurement. Thus the first post-stimulation measurement started 20 min after the end of tDCS. A second, faster 3D T1-weighted acquisition was performed in order to place the spectroscopy voxel in the position identical with that of pre-stimulation. After shimming, ten consecutive post-stimulation GABA measurements were sampled at every 6 minutes interval for approximately sixty-four minutes (*post-stimulation* measurements). Correct placement of voxels after tDCS was confirmed using a screenshot of the voxel location taken before stimulation measurements. A graphical description of the experimental layout is depicted in Fig. 3. The participants were asked to keep their eyes open during the whole experimental procedure and were informed about the start and duration of each measurement before acquisition.

All GABA concentration levels as shown in Table 1 were normalized to the first baseline pre-stimulation measurement before running statistical analyses. The second pre-stimulation measure served as control for spontaneous fluctuations of GABA concentration irrespective of experimental manipulations. To verify that the voxels of interest (VOI) were equivalent pre- and post-tDCS, a set of mixed ANOVAs was conducted to examine any changes in grey and white matter fractions (as shown in Table 2) within each VOI. The ANOVA included the within-subject factor time (pre- vs. post-tDCS) and the between-subject factor tDCS polarity (anodal vs. sham). The analyses confirmed that in all cases there were no significant main or interaction effects (all p > 0.05). These analyses confirm that tissue fractions pre- and post-tDCS did not differ.

**Table 1:** Mean GABA concentrations related to anodal and sham tDCS.

	Measurements	Mean_GABA Conc [mM( ± SD)] (Anodal group)	Mean_GABA Conc [mM( ± SD)] (Sham group)
Before stimulation	1	1.68(±0.34)	1.76(±0.19)
	2	1.67(±0.29)	1.67(±0.18)
After Stimulation	1	1.47(±0.45)	1.76(±0.23)
	2	1.53(±0.42)	1.71(±0.25)
	3	1.61(±0.34)	1.66(±0.20)
	4	1.50(±0.48)	1.68(±0.25)
	5	1.49(±0.44)	1.66(±0.24)
	6	1.53(±0.46)	1.72(±0.24)
	7	1.53(±0.52)	1.70(±0.22)
	8	1.49(±0.45)	1.65(±0.22)
	9	1.48(±0.48)	1.70(±0.31)
	10	1.42(±0.45)	1.71(±0.27)

**Table 2:** Mean Grey Matter, White Matter and CSF values.

	Subjects	Grey Matter	White Matter	CSF
Mean(±SD)	32	0.45(±0.08)	0.42(±0.05)	0.13(±0.08)

### MRS data analysis

**Figure 4:**
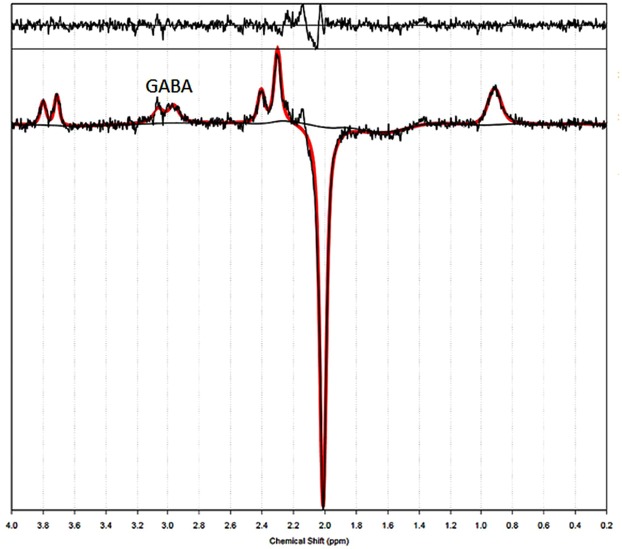
An edited spectrum from a single subject shows characteristic peaks for GABA after application of a linear combination model using TARQUIN to perform a fully automatic fit of spectra. The acquired spectrum is plotted in black and the fit in red. Below and above the acquired spectrum are the baseline and residual shown respectively.

The freely available software package TARQUIN (Totally Automatic Robust Quantitation in NMR, version 4.3.5) was used to quantify RDA files containing MRS data. This tool utilizes a linear combination of basis functions to perform a fully automatic analysis of spectra^49^ and its reliability is comparable with other spectral quantification methods (O’Gorman *et al*., 2011). The free induction decay signal (FID) was zero filled to twice its original length to obtain reference at a higher precision. The residual water signal was then filtered out by fitting and removing Gaussian peaks around the water frequency using hankel singular value decomposition techniques. The spectrum was then frequency and phased corrected with respect to both the zero- and first-order phase. To analyze MEGA-PRESS data, TARQUIN models the GABA peak as composed by two single Gaussians^46^. The unsuppressed water scan was used as internal reference to find the absolute value of GABA concentration. The 3-ppm GABA peak in the difference spectrum was fitted using a two single Gaussian and quantified relative to water. The final spectral quality was assessed using Cramér-Rao-Lower-Bounds^50^ minimum possible variance on a fit parameter. Only data (as shown in Fig. 4) that had Cramér-Rao-Lower-Bounds values of less than ≤20% were included in the analysis. The concentration of GABA was corrected for the proportion of grey matter within the voxel using the MPRAGE anatomical image^13^. The voxel fraction of CSF, gray matter, and white matter were calculated after generation of a binary mask of the MRS voxel created with the same imaging matrix as the T1-weighted anatomical image, using Gannet’s^51^ integrated voxel-to-image coregistration and segmentation of the anatomical image using Segment in SPM12^52^.

## Data Availability

The datasets generated during and/or analyzed during the current study are freely available from the open access *OSF* repository (https://osf.io/*)*. https://osf.io/bkzey/?view_only=320b254a563b4b5d8cd9c6891a6d110a.
